# The Standard Dictionary of Funk & Wagnall’s

**Published:** 1894-08

**Authors:** 


### The Standard Dictionary of Funk & Wagnalls, which was reviewed in this journal, April number, page 122, has been constructed with especial reference to avoiding the recognition of needless new literary terms—words coined by the caprice or mistaken judgment of this or that author. A committee of representative scholars has passed upon new literary words before they were admitted into the Dictionary. The judgment of the scientific specialists has determined the admission or rejection of technical terms. Upon their decision, not a few technical terms recorded in other dictionaries have been rejected, some because they are obsolete, and others because they are so rarely used as to be comparatively valueless. This simple general rule of inclusion has been followed: *Omit no word found in a living book*—that is, in a book now read by any considerable number of people—and whose meaning is likely to be sought for in an English dictionary. (See *Introductory*, Vol. I, p. viii.)

In the definitions, special pains has been taken to make the work as thorough as possible, by presenting exact and recent meanings and distinctions, by giving a definite clue to the great departments and divisions of knowledge, and by making the general definitions comprehensive and exhaustive.

We ask attention to a few typical instances illustrating the quality and method of definition and distribution: (1) Distinctions heretofore overlooked or inadequately presented; as, *accessary* and *accessory; acclimate* and *acclimatize; affect*[1] and *affect*[2]*; affection*[1] and *affection*[2]*; corelative, corelation, corelatively*, and *correlative, correlation, correlatively; cyclian* and *cyclic*. (2) Distribution of Sciences—*anthropology, architecture, botany, cosmology, esthetics, idealism, language, literature*. (3) General Definitions—*absolute, age, agnosticism, animal, antecedent, arithmetic, art, atonement, cause, choral, construction, cross, drama, division, electricity, energy, force, genius, gnosiology, good, imagination, induction, intuition, judgment, know, knowledge.*

